# Acute aluminium phosphide poisoning: Can we predict mortality?

**DOI:** 10.4103/0019-5049.68372

**Published:** 2010

**Authors:** Ashu Mathai, Madhurita Singh Bhanu

**Affiliations:** Department of Anaesthesiology & Critical Care, Christian Medical College and Hospital, Ludhiana, Punjab, India

**Keywords:** Aluminium phosphide, mortality, poisoning

## Abstract

In India, acute aluminium phosphide poisoning (AAlPP) is a serious health care problem. This study aimed to determine the characteristics of AAlPP and the predictors of mortality at the time of patients’ admission. We studied consecutive admissions of patients with AAlPP admitted to the intensive care unit (ICU) between November 2004 and October 2006. We noted 38 parameters at admission to the hospital and the ICU and compared survivor and non-survivor groups. A total of 27 patients were enrolled comprising5 females and 22 males and the mean ingested dose of poison was 0.75 ± 0.745 grams. Hypotension was noted in 24 patients (89%) at admission and electrocardiogram abnormalities were noted in 13 patients (48.1%). The mean pH on admission was 7.20 ± 0.14 and the mean bicarbonate concentration was 12.32 ± 5.45 mmol/ L. The mortality from AAlPP was 59.3%. We found the following factors to be associated with an increased risk of mortality: a serum creatinine concentration of more than 1.0 mg % (*P* = 0.01), pH value less than 7.2 (*P* = 0.014), serum bicarbonate value less than 15 mmol/L (*P* = 0.048), need for mechanical ventilation (*P* = 0.045), need for vasoactive drugs like dobutamine (*P* = 0.027) and nor adrenaline (*P* = 0.048) and a low APACHE II score at admission (*P* = 0.019). AAlPP causes high mortality primarily due to early haemodynamic failure and multi-organ dysfunction

## INTRODUCTION

Acute aluminium phosphide poisoning (AAlPP) is a large, though under-reported, problem in the Indian subcontinent. Aluminium phosphide is reported to be highly toxic when consumed from a freshly opened container and the fatal dose for an average sized individual is believed to be 150 to 500 grams.[1] Death is reported to result from profound shock, myocarditis and multiorgan failure.

The mortality rates from AAlPP published in literature vary from 40-80%.[1] However, the actual numbers of cases affected are much larger, as less than 5% of those with AAlPP eventually reach a tertiary care centre.[2] Since 1992, when aluminium phosphide became freely available in the market, it has reportedly, overtaken all other forms of deliberate poisoning like organophosphorus and barbiturate poisoning in northern India.[3] In their 25 year study on 5,933 unnatural case fatalities in north-west India by Singh D *et al*., AAlPP was the major cause of death among all poisonings.[4]

Despite these large numbers, there has been little progress in our understanding of the characteristics of the poison and limited Indian data is available on the predictors of mortality in these patients. The purpose of this study was to retrospectively study the profile of patients presenting with AAlPP and to identify the factors at admission that might be useful in predicting mortality.

## METHODS

### Study design

All consecutive cases of AAlPP presenting to a tertiary care hospital in northern India over a two-year period from November 2004 to October 2006 were retrospectively reviewed. The diagnosis of AAlPP was based on history of consumption of the poison (obtained from the patient or the closest relative) and symptomatology at admission. Instances where the patients had presented with an unclear diagnosis of poisoning or where there was consumption of more than one substance were excluded from the study.

All patients were admitted into our ICU after initial resuscitation and gastric lavage. A baseline electrocardiogram was recorded and blood samples for biochemical and haematological investigations were sent from the emergency room within an hour of presentation to the hospital. Infusion of vasoactive agents and mechanical ventilatory support were instituted where indicated. We collected 38 variables, like age, gender, nature of poisoning (suicidal/accidental), the dose and characteristics of the poison consumed (exposed/unexposed form) and the delay in presentation to hospital. The nature of any first aid instituted before reaching this hospital as well as laboratory parameters on admission to the hospital and ICU were also noted. The severity of the poisoning was assessed from the extent of organ dysfunction (renal, hepatic, neurological, gastrointestinal, cardiovascular, etc.), the need for mechanical ventilation and the requirement of drugs for vasoactive support. We also calculated severity of illness scores like APACHE II (Acute Physiology and Chronic Health Evaluation) and SAPS II (Simplified Acute Physiology Score) on all patients based on the data available [Table 1].

**Table 1:** Variables collected on patients presenting with aluminium phosphide poisoning

Baseline parameters	
Name	Age/Sex unit number
Occupation	Time delay after ingestion
Intent	Characteristics of poison -exposed/unexposed
Dose consumed	Additives, if any
Symptoms	Signs
Gastrointestinal	Glasgow coma scale
Cardiac	Cardiac
Respiratory	Respiratory
Neurological	Gastrointestinal
Laboratory investigations	
paO_2_	Serum sodium
paCO_2_	Serum potassium
Serum creatinine	Serum blood sugar level
pH	Serum bicarbonate
Serum bilirubin	AST/ALT
Treatment given	
Use of mechanical ventilation/non-invasive ventilation	
Use of vasoactive drugs	Magnesium therapy
Dopamine	Steroid therapy
Dobutamine	
Nor-adrenaline	
Scores calculated	
APACHE II score	SAPS II score

### Statistical analysis

The SPSS computer program was used to process the data and generate the statistics. Univariate analysis was performed to compare survivors with non survivors groups (*P* < 0.05 was considered significant).

## RESULTS

**Figure 1:**
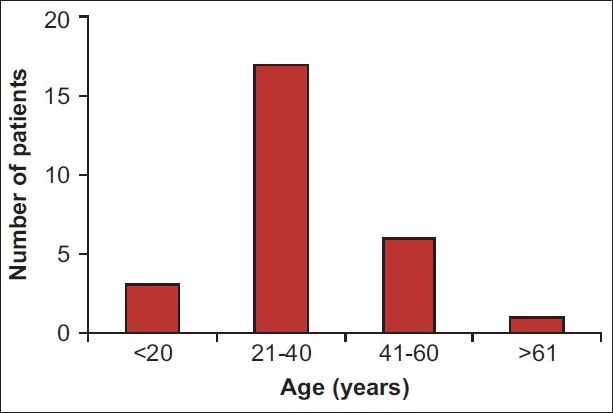
Age distribution of patients with acute aluminium phosphide poisoning. The majority of victims were young, in the age group of 21 to 40 years

A total of 27 patients with AAlPP were admitted into our ICU during the study period. The majority of patients were young and in the age group from 21 to 40 years [Figure 1] with males outnumbering females by more than 4:1.

Most of the cases involved suicidal consumption of the poison (92%) and 60% of the poison was consumed in the unexposed form of the tablet and an average of 1.53 grams of drug was consumed. There was a mean delay of 2.1 ± 1.55 hours before presenting to this hospital. There was no significant association between the dose of poison consumed or the time delay in presentation to the hospital with mortality. We also did not find any association between consumption of the poison in its unexposed form and mortality (*P* = 0.922) [Table 2].

**Table 2:** Demographic profile: The demographic profile of patients presenting with acute aluminium phosphide poisoning were comparable between survivors and nonsurvivors

Variables	Survivors	Non-survivors	*P*
	(n = 11)	(n = 16)	value
	Mean ± 1 SD*	Mean ± 1 SD*	
	or n†	or n†	
Age	29.25 ± 14.119	35.5 ± 10.488	0.206
Sex (male/female)	9/2	13/3	0.970
Time delay (hours)	2.00 ± 1.543	2.29 ± 1.607	0.643
Dose consumed (grams)	1.64 ± 1.027	1.47 ± 0.498	0.581
Unexposed/exposed form	7/1 (3 - unknown)	9/2 (5 - unknown)	0.922
Self poisoning	10	15	-

* SD - Standard deviation

† n- number of patients

At presentation to the hospital, the most predominant feature experienced by patients was vomiting and nausea (92.6%). A few patients had respiratory distress (7%). The mean Glasgow coma scale at admission was 13.29 ± 2.825 and the mean partial pressure of oxygen in arterial blood (paO_2_) and partial pressure of carbon dioxide in arterial blood (paCO_2_) in patients were 72.63 ± 4.06 mm Hg and 26.37 ± 7.46 mm Hg, respectively. A total of 7 patients (25%) had high serum creatinine values at admission, all of whom eventually died. Serum creatinine levels were found to correlate well with mortality. Survivors had significantly lower serum creatinine levels at admission as compared to non-survivors (0.82 ± 0.1418 milligram per deciliter versus 1.375 ± 0.642 milligram per deciliter respectively, *P* = 0.011). The mean pH of patients at admission too, was a good indicator of prognosis. Survivors had a much higher average value (7.284 ± 0.151) than non-survivors (7.148 ± 0.120) and this difference was statistically significant (*P* = 0.015). Similarly, serum bicarbonate levels at admission also correlated well with the eventual outcome in these patients (*P* = 0.048). All patients had normal levels of sodium at admission to the hospital while 48% of patients had hypokalemia. These variables were statistically insignificant. Increased serum levels of bilirubin, aspartate aminotranferase, alanine aminotransferase and random blood sugar at admission did not show any association with mortality. The salient clinical investigations noted at admission are as given in Table 3.

**Table 3:** Relevant clinical investigations at admission in patients with acute aluminium phosphide poisoning

Variable	Mean ± SD*	Range
GCS† (score)	13.29 ± 2.825	3-15
PaO_2_‡ (mm of Hg)	72.63 ± 4.06	42-90
PaCO_2_§ (mm of Hg)	26.37 ± 7.46	14-42
Serum creatinine (mg %)║	1.68 ± 1.89	0.6-2.3
ALT (U/L)¶ (U/L)**	38.52 ± 10.58	26-70
AST†† (U/L)**	43 ± 11.21	30-78
Serum sodium (mg %)║	139.04 ± 3.20	129-147
Serum potassium (mg %)║	3.59 ± 0.67	2.6-4.5
Serum bilirubin (mg %)║	0.77 ± 1.08	0.03-0.9
Blood sugar (mg %)║	163.11 ± 37.17	120-240
pH	7.204 ± 0.147	6.801-7.512
Serum bicarbonate (mmol/L)‡‡	12.32 ± 5.46	3.9-29
APACHE ║§§ score	12.148 ± 6.608	3-29
SAPS ║║║ score	31.444 ± 14.781	4-65

* SD - Standard deviation

† GCS - Glasgow coma scale

‡ paO_2_ - Partial pressure of oxygen in arterial blood

§ paCO_2_ - Partial pressure of carbon dioxide in arterial blood

║ mg % - milligram per deciliter

¶ ALT - Alanine aminotransferase

** U/L - Units per litre

†† AST - Aspartate aminotransferase

‡‡ mmol/L - millimoles per litre

§§ APACHE- Acute physiology and chronic health evaluation score

║║ SAPS - Simplified acute physiology score

**Figure 2:**
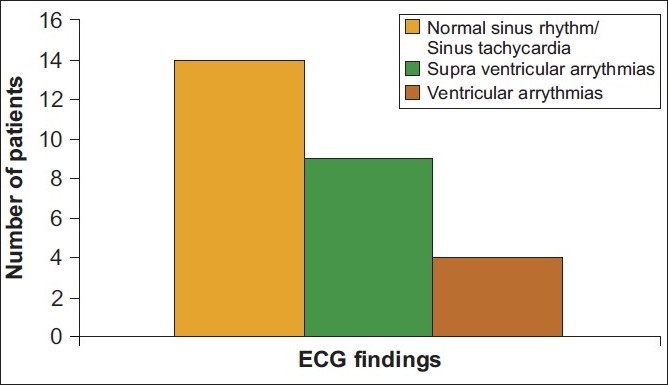
ECG abnormalities at admission. Most of the dysrhythmias were supraventricular. Presence of dysrhythmias showed a trend towards increasing mortality

We found that 81% of patients had cardiac symptoms, mainly in the form of hypotension and/or arrhythmias on admission to the hospital. A total of 13 patients had dysrhythmias at admission, of which, the majority (69%) were of supraventricular origin [Figure 2]. Though the presence of electrocardiographic abnormalities did not predict mortality, there was a trend towards increasing mortality in patients with dysrhythmias (*P* = 0.07).

Immediately on admission into the intensive care unit, 50% of the patients required mechanical ventilatory support while non invasive ventilation was used only in one patient. Eighty nine percent of the patients were in shock at admission despite adequate fluid resuscitation and needed vasoactive support, predominantly dobutamine and nor adrenaline. In all patients, magnesium sulphate was used for treatment while systemic steroid therapy (injectable hydrocortisone) was initiated in 70% of patients (mainly depending on the treating physician).

**Figure 3:**
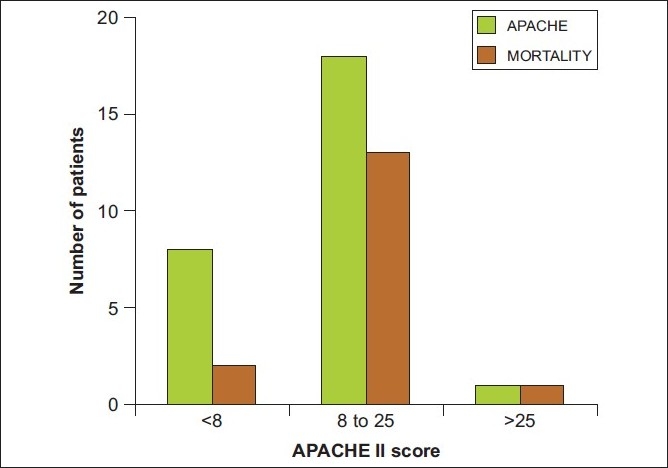
Correlation of APACHE II score with mortality. The APACHE II score predicted mortality with good accuracy. The scores were significantly different between survivors and non-survivors. In patients with APACHE II scores of more than 8, mortality was 73%

**Figure 4:**
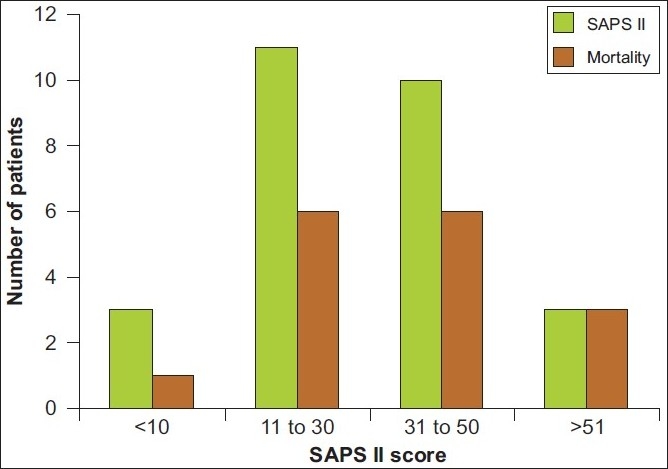
Correlation of SAPS II score with mortality. Though the SAPS II score did not show a significant difference between survivors and non-survivors, in patients with SAPS II score of more than 30, the mortality rate was 69.2%

The mean APACHE II score at admission to the hospital was 12.14 ± 6.608 and SAPS II score was 31.44 ± 14.781 [Figures 3 and 4]. We found that, in patients with APACHE II scores of more than 8, the rates of mortality were 73% and in patients with SAPS II scores of more than 30, the mortality rates were 69.2%. The APACHE II score at admission correlated well with mortality and survivors had significantly lower score than non-survivors (score 8.64 ± 5.27 vs 14.56 ± 6.66, respectively, *P* = 0.019). The SAPS II score did not show the same degree of significance, though the average score was higher in non-survivors. (26.36 ± 13.66 versus 34.94 ± 15.58 in survivors and non-survivors respectively, *P* = 0.142) [Table 4].

**Table 4:** Factors evaluated for prediction of mortality in patients with acute aluminium phosphide poisoning (univariate analysis)

Variables	Survivors	Non-survivors	*P*
	(n = 11)	(n = 16)	value
	Mean ± SD or n*	Mean ± SD or n*	
APACHE║† (score)	8.64 ± 5.27	14.56 ± 6.66	0.019
SAPS║‡ (score)	26.36 ± 13.66	34.94 ±15.58	0.142
ECG§ abnormalities	3/11	10/16	0.072
Hypotension ± arrhythmias	7/11	15/16	0.085
pH	7.284 ± 0.151	7.148 ± 0.120	0.015
pH < 7.2	3/11 11/16 0.014
Se║ bicarbonate (mmol/L)¶	15.24 ± 6.89	10.32 ±3.46	0.048
Se creatinine (mg %)**	0.82 ± 0.1418	1.375 ± 0.642	0.011
Se sodium (mg %)**	140 ± 2.79	138.37 ± 2.82	0.782
Se potassium (mg %)**	3.68 ± 0.62	3.53 ± 0.72	0.816
Blood sugar (mg %)**	157.1 ± 43.24	167.25 ± 35.64 0.310
Mechanical ventilation	4/11	11/16	0.045
Vasoactive drugs	8/11	16/16	0.027
1) Dopamine	6/11	14/16	0.055
2) Dobutamine	8/11	9/16	0.048
3) Nor- Adrenaline	2/11	9/16	0.048
Magnesium therapy	11/11	16/16	-
Steroid therapy	8/11	11/16	0.824

* n - number of patients

║ Se - Serum

† APACHE - Acute physiology and chronic health evaluation score

‡ SAPS - Simplified acute physiology score

§ ECG - Electrocardiogram

¶ mmol/L - millimoles per litre

** mg% - milligram per deciliter

*P* < 0.05 is considered significant.

The overall mortality from AAlPP poisoning in our unit during the study period was 59.3%. The statistically significant factors useful in predicting mortality in our study were, an elevated serum creatinine concentration (*P* = 0.01) at admission, need for mechanical ventilation (*P* = 0.045), need for vasoactive drugs like dobutamine (*P* = 0.027) and nor adrenaline (*P* = 0.048), pH value less than 7.2 (*P* = 0.014), a low serum bicarbonate value (*P* = 0.048) and a high APACHE II score (*P* = 0.019).

## DISCUSSION

Aluminium Phosphide is an extremely toxic compound and resulted in a high mortality rate of 59.3% in our study. The toxicity of Aluminium Phosphide is attributed to the liberation of phosphine gas which is cytotoxic and causes free radical mediated injury.

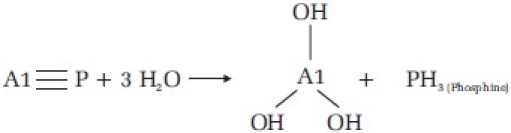



Phosphine a nucleophile, acts as a strong reducing agent capable of inhibiting cellular enzymes involved in several metabolic processes. Early studies on phosphine demonstrated specific inhibitory effects on mitiochondrial cytochrome c oxidase.[5] Experimental and observational studies have subsequently demonstrated that the inhibition of cytochrome c oxidase and other enzymes leads to the generation superoxide radicals and cellular peroxides. Cellular injury subsequently occurs through lipid peroxidation and other oxidant mechanisms.[6,7] Chugh *et al*. reported that, serum phosphine levels correlate positively with the severity of poisoning and levels equal to or less than 1.067 ± 0.16 mg % appear to be the limit of phosphine toxicity.[8] The major lethal consequence of aluminium phosphide ingestion i.e., profound circulatory collapse, is reportedly secondary to these toxins generated, which lead to direct effects on cardiac myocytes, fluid loss, and adrenal gland damage.[9] In addition, phosphine also has corrosive effects on tissues.[9]

As less than 5% of patients with AAlPP eventually reach a tertiary care hospital, it is extremely difficult to know the actual incidence of these poisonings in our country and it is postulated that the spectrum of actual cases may be much larger than that reported. In one study of 559 cases of all acute poisonings presenting over 14 months, aluminium phosphide was found to be the commonest poison consumed (79.8%) in the Haryana- Rohtak belt with a mortality of 67.6%.[10]

In our study we found that most of the victims were young with males outnumbering females. As with other poisonings, aluminium phosphide is a common method of attempting suicide among the younger, productive age group of society. The patients in our study typically developed gastrointestinal symptoms early in their presentation and the predominant early toxic manifestation was cardiac (hypotension and arrhythmias). Most patients needed some form of vasopressor/ionotrope support and the need to use these early after admission was clearly associated with a poorer outcome.

In their study on 418 patients, with aluminium phosphide poisoning over seven years, Chugh *et al*. reported gastrointestinal symptoms as the commonest while conduction disturbances and arrhythmias occurred in 38% of patients and an overall mortality rate of 77%.[11]

The management of AAlPP remains purely supportive. Though magnesium sulphate was used in the treatment of all our patients, there is conflicting data in literature on the role of magnesium sulphate in the treatment of acute aluminium phosphide poisoning. Some studies seem to suggest that there is hypomagnesaemia associated with AAlPP and that there is a direct relationship between abnormal electrocardiographic findings and low magnesium levels. They report reduced mortality rates with magnesium therapy in these patients.[12,13] However other studies have shown no such benefits and some have even demonstrated hypermagnesaemia in patients with AAlPP.[14] In their study on the role of magnesium in such patients, Siwach *et al*., reported that there was no evidence of hypomagnesaemia in these patients nor did magnesium sulphate therapy improve survival.[15]

We found six factors that can be assessed at admission to the hospital to predict mortality from aluminium phosphide ingestion. These include an elevated serum creatinine concentration, a low pH value (less than 7.2), a low serum bicarbonate value (less than 15), a low APACHE II score, an early need for mechanical ventilation and for vasoactive drugs like dobutamine and nor adrenaline for haemodynamic support. These findings are similar to that obtained by Louriz *et al*., who concluded that the prognostic factors associated with mortality from AAlPP, included a low APACHE II score, low Glasgow coma scale score, shock, electrocardiogram abnormalities, the presence of acute renal failure, low prothrombin rate, hyperleukocytosis, use of vasoactive drugs and use of mechanical ventilation.[16] In a recent update on AAlPP by Wahab, *et al*., the development of refractory shock, ARDS, aspiration pneumonitis, anaemia, metabolic acidosis, electrolyte imbalance, coma, severe hypoxia, gastrointestinal bleeding, and pericarditis were the factors reportedly associated with poor prognosis. They also noted that the outcome from AAlPP correlates best with the number of vomiting the patient gets after ingestion and the severity of hypotension that the patient develops.[17]

The limitation of our study was that it was retrospectively designed. Hence, larger prospective studies need to be done in the future to conclusively support our results.

We conclude that aluminium phosphide is an extremely toxic compound with mortality close to 60%. The variables at admission which could be used to detect patients at greater risk of mortality from AAlPP include, need for mechanical ventilation, hypotension at admission requiring vasoactive drugs, low pH values, low serum bicarbonate levels, low serum creatinine levels and low APACHE II scores.
